# Artificial oocyte activation with Ca^2+^ ionophore improves reproductive outcomes in patients with fertilization failure and poor embryo development in previous ICSI cycles

**DOI:** 10.3389/fendo.2023.1244507

**Published:** 2023-08-11

**Authors:** Jing Ling Ruan, Shan Shan Liang, Jia Ping Pan, Zhi Qin Chen, Xiao Ming Teng

**Affiliations:** ^1^ Reproductive Medicine Center of Shanghai First Maternity and Infant Hospital, School of Life Sciences and Technology, Tongji University, Shanghai, China; ^2^ Shanghai Institute of Maternal-Fetal Medicine and Gynecologic Oncology, Shanghai First Maternity and Infant Hospital, School of Medicine, Tongji University, Shanghai, China

**Keywords:** artificial oocyte activation, ionomycin, intracytoplasmic sperm injection, fertilization failure, embryo development

## Abstract

**Research question:**

Does artificial oocyte activation (AOA) by a calcium ionophore (ionomycin) improve the previous fertilization failure or poor embryo development of intracytoplasmic sperm injection (ICSI) account for male factor infertility or other infertility causes?

**Design:**

This retrospective study involved 114 patients receiving ICSI-AOA in Shanghai First Maternity and Infant Hospital with previous ICSI fertilization failure or poor embryo development. The previous ICSI cycles of the same patients without AOA served as the control group. The fertilization rates, cleavage rates, transferable embryo rates and blastocyst formation rates of the two groups were compared. Additionally, the clinical pregnancy, implantation rate and live birth rates were also compared to assess the efficiency and safety of AOA. Furthermore, two subgroup analyses were performed in this study based on the cause of infertility and the reason for AOA. The fertilization rate, embryonic development potential and clinical outcome were compared among groups.

**Results:**

Among 114 ICSI-AOA cycles, the fertilization rate, top-quality embryo rate, implantation rate, clinical pregnancy per patient and live birth rate per patient were improved significantly compared with previous ICSI cycles (p<0.05 to P< 0.001), and the miscarriage rate in the AOA group was significantly lower than that of the control group (p<0.001). In the AOA subgroups based on the cause of infertility, the fertilization rates of each subgroup were significantly improved compared with previous control cycles except for the mixed factor infertility subgroup (p<0.05 to p<0.001). In the AOA subgroups based on the reason for AOA, the fertilization rates of each subgroup were significantly increased compared with those in their previous ICSI cycle without AOA (p<0.001); however, there was no significant difference in the top-quality embryo rate. No significant improvement was found in the implantation rates and the clinical pregnancy rate in each subgroup except for the poor embryo development subgroup. In the 114 AOA cycles, 35 healthy infants (21 singletons and 7 twins) were delivered without major congenital birth defects or malformations.

**Conclusion:**

This study showed that AOA with the calcium ionophore ionomycin can improve the reproductive outcomes of patients with previous fertilization failure and poor embryo development after ICSI.

## Introduction

Intracytoplasmic sperm injection (ICSI) is mainly used for male factor infertility. The average fertilization rate after ICSI is approximately 70% to 80% (1). However, 1%~5% of ICSI cycles occurring still result in total fertilization failure (TFF) or almost complete fertilization failure, defined as a fertilization rate of less than 30% (2). Oocyte activation deficiency (OAD) is the main cause of ICSI fertilization failure, which may be related to sperm or oocyte factors leading to responsiveness of gamete interaction (3, 4).

In the past decade, an increasing number of studies have shown that sperm-specific phospholipase Cζ (phospholipase C zeta, PLCζ) is strongly associated with sperm-specific oocyte activation failure (OAF) (5). The low expression of PLCζ is one of the main reasons for fertilization failure (6–8). ICSI combined with artificial oocyte activation could mimic physiological calcium changes that occur during fertilization (9). During normal fertilization, sperm-specific PLCζ is released into the oocyte cytoplasm, triggering calcium shock and promoting the release of Ca^2+^ in the endoplasmic reticulum. The temporary increase of Ca^2+^ concentration in oocytes generates Ca ^2+^ oscillations to activate oocytes and induces a cortical granular response. Oocytes recover and complete meiosis, extrude the second polar body and form two pronuclei (8). The endoplasmic reticulum is the main source of intracellular Ca^2+^ increase, which is mainly triggered by the successful induction of IP3 pathway by sperm-specific phospholipase C-ζ (PLC-ζ) around the sperm nucleus. The failure of oocyte activation leading to non-fertilization may be mainly caused by the failure of PLC-induced IP3 pathway (10). However, the molecular mechanism of OAD induced by oocyte-borne oocyte activation factors is unclear. According to previous reports, human oocytes with ICSI-TFF often carry with specific problems, such as abnormal cell structure, abnormal gene expression, abnormal chromosomes, immature oocytes and abnormal spindles (11). All of these problems may cause oocyte activation failure after ICSI.

AOA is the most commonly used technology to solve sperm-related OAF at present, and healthy live births have been delivered after using various activation methods. Abnormalities in the oocyte activating signal pathway may lead to the occurrence of OAF (12). To date, AOA is the most effective method to solve ICSI-TFF, low fertilization and OAF caused by sperm factors (13). However, large-scale clinical studies are still lacking, and there are also disputes regarding the efficiency of AOA and the safety of offspring. Therefore, this study analysed the clinical pregnancy outcomes and neonatal health of AOA patients who received the Ca^2+^ ionophore ionomycin during ICSI and evaluated the efficacy and safety of ionomycin on the fertilization rate and embryo development.

## Materials and methods

### Patients

**Figure 1:**
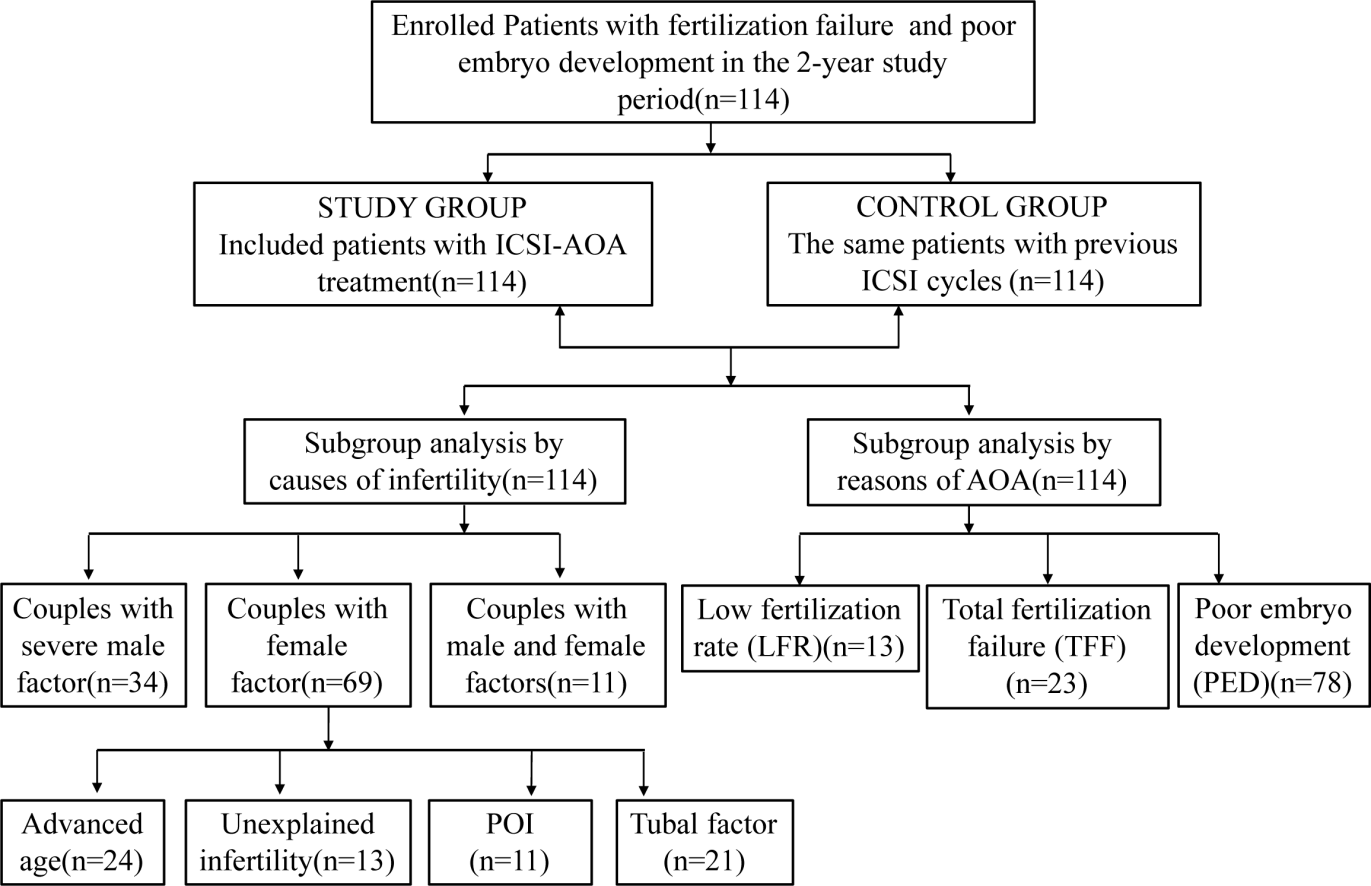
Flowchart of patients. ICSI, intracytoplasmic sperm injection; AOA, artificial oocyte activation; POI, patients with primary ovarian insufficiency.

This retrospective analysis was performed on the clinical data of couples receiving ICSI-AOA due to poor fertilization or poor embryo development after a previous ICSI cycle from January 2019 to December 2020 in Shanghai First Maternity and Infant Hospital affiliated with Tongji University. The indications for ICSI-AOA are as follows: previous ICSI fertilization failure (TFF or fertilization rate ≤ 30%) and poor embryo development (complete embryo development arrest, reduced blastocyst formation on day 5 (≤15%)). Controlled ovarian stimulation and oocyte retrieval were carried out according to standard protocols (14). A total of 114 couples were enrolled in this retrospective analysis (**Figure 1**). Most patients started ICSI with ovarian stimulation using either the long agonist or antagonist protocols. Ovulation was triggered by intradermal injection of 5000~10,000 IU HCG. Patients with AOA cycles in the previous cycle and half-AOA cycles were excluded from this study. The previous cycles without AOA were considered as a control group. The interval between the two stimulation cycles was no more than six months.

In addition, two subgroup analyses were performed in this study. According to the cause of infertility, patients were further divided into six subgroups: (i) oligoasthenoteratozoospermia (OAT), including oligozoospermia (low number of sperm), asthenozoospermia (poor sperm movement), and teratozoospermia (abnormal sperm shape); (ii) advanced age (age>35 years); (iii) primary ovarian insufficiency (POI, patients with primary ovarian insufficiency); (iv) unexplained infertility; (v) tubal factor; and (vi) mixed factor infertility (M+F). Based on the reason for AOA, patients were divided into three subgroups for analysis: total fertilization failure (TFF); low fertilization rate (LFR, fertilization rate <30%); and poor embryo development (PED). Similarly, the oocyte activation cycle was compared with the previous ICSI cycle of the same patients. The fertilization rate, embryonic development potential and clinical outcome were compared among groups.

This study was approved by the institutional review board of Shanghai First Maternity and Infant Hospital affiliated with Tongji University. All the enrolled patients in this study signed written informed consent and were informed of the potential risks of using AOA technology.

### ICSI and calcium ionophore AOA procedure

The oocytes collected from each cycle were cultured at 37°C in a humidified triple-gas incubator with 6% CO_2_ and 5% oxygen (Thermo Fisher Scientific, USA) for at least 2 h before removing cumulus cells. Subsequently, all metaphase II oocytes were treated according to the standard ICSI procedure (15). Oocyte activation with ionomycin was performed according to previous reports (16, 17). Briefly, thirty minutes after ICSI, the oocytes were immediately exposed to activated liquid droplets (G-mops, Vitrolife, Sweden) containing 10 μmol/l ionomycin (Sigma Aldrich, USA) for 10 minutes, rinsed with medium (G-mops, Vitrolife, Sweden) 3 times, and transferred to embryo culture medium (G1, Vitrolife, Sweden).

### Fertilization check, embryo grading and embryo transfer

Fertilization observations were carried out 18-19 h after ICSI under an inverted microscope. Only oocytes with two clear pronuclei (2PN) and two polar bodies (2PB) were considered to be fertilized normally. Quality assessment of embryos was carried out according to routine guidelines described previously (18). Embryos were cultured *in vitro* to the third day (D3), and only embryos with 7-9 cells from normal fertilized oocytes and a fragment ratio <10% were defined as top-quality embryos. Then, top-quality embryos were transferred under transvaginal ultrasound guidance. The remaining top-quality embryos were cryopreserved for the next cycle. Additionally, all the remaining cleavage-stage embryos were cultured up to the blastocyst stage, the blastocysts were scored morphologically according to the Gardner scale (2), and the available blastocysts were cryopreserved for later transfer. According to provincial guidelines, up to two embryos can be transferred in any transfer cycle.

### Study outcome assessment and clinical pregnancy

The following parameters were used to compare previous routine ICSI cycles with ICSI-AOA cycles of the same patient. The primary outcome measure was the 2PN fertilization rate, defined as the number of normal fertilized oocytes divided by the number of injected MII oocytes. Secondary outcome measures included the cleavage rate (number of cleaved zygotes/number of fertilized oocytes), top-quality embryo rate (number of high-quality embryos/number of cleaved zygotes), blastocyst formation rate (number of blastocysts formed/number of blastocysts cultured), and implantation rate (number of gestational sacs seen on scanning divided by the number of embryos replaced). Clinical pregnancy was defined as the presence of at least one gestational sac on ultrasound at 6 weeks. A baby born alive after 20 gestation weeks was classified as a live birth. The miscarriage rate was defined as the number of miscarriages before 20 weeks divided by the number of women with a positive pregnancy test. Additionally, information on the patient’s pregnancy outcomes was followed up until March 2021 by telephone survey, including gestation weeks, gender of babies, birth weight and body length, as well as major birth defects. There were still 40 and 103 frozen embryos remaining in the previous ICSI cycles and the AOA cycles, respectively, by the end of the study.

### Statistical analysis

The one-sample Kolmogorov–Smirnov test was used to test the normal distribution of continuous variables. Continuous variables were given as the mean ± SD if normally distributed. Comparisons between groups were performed with paired t tests for continuous variables and chi-square tests for categorical variables, where appropriate. Statistical analysis was performed using the Statistical Program for Social Sciences (SPSS Inc., Version 14.0, Chicago, IL, USA). A two-tailed value of P < 0.05 was considered statistically significant.

## Results

### Patients’ baseline clinical characteristics

Between January 2019 and December 2020, 114 patients who underwent ICSI-AOA were enrolled in this study with a history of poor fertilization or embryo development in the first ICSI cycles. The flow chart of the subjects is shown in **Figure 1**.

The average age of the women was 33.86 ± 4.981 years, and they had suffered infertility for 4.09 ± 2.991 years before ART treatment (**Table 1**). The other baseline characteristics of the patients are shown in **Table 1**, including infertility types, infertility years, infertility factors, female body mass index (BMI), antral follicle counts (AFC), and basal FSH. All couples received routine ICSI and AOA (**Table 1**). The stimulation protocol used in each group was similar between the two groups (P >0.05) (**Table 2**).

**Table 1:** General characteristics of the enrolled patients (mean ± SD).

Item	Value
No. of patients	114
Female age (y), mean ± SD	33.86 ± 4.981
Male age (y), mean ± SD	35.71 ± 5.799
Type of infertility, n (%)	
Primary infertility	72 (63.16%)
Secondary infertility	42 (36.84%)
Duration of infertility (y)	4.09 ± 2.991
Indication, n (%)	
Female factors	68(59.65%)
Male factors	35(30.70%)
Mixed	11(9.65%)
BMI for women (kg/m^2^)	22.29 ± 3.291
AFC	9.99 ± 7.378
Basal FSH(IU/L)	7.24 ± 3.923

Values are shown as the mean ± SD or number. BMI, female body mass index; AFC, antral follicle count.

**Table 2:** Comparison of clinical outcomes previous standard ICSI cycles and AOA cycles.

	Control group (before)	AOA group (after)	*t/χ ^2^/Z* value	*P* value
No. of patients	114	114		
COS protocols				0.648
long protocol (%)	59(51.75)	66(57.89)	0.868	
Antagonist protocol (%)	40(35.09)	35(30.70)	0.497	
Other protocol (%)	15(13.16)	13(11.40)	0.163	
No. of oocytes obtained	6.89 ± 4.450	7.75 ± 5.048	1.830	0.070
No. of MII oocytes	5.33 ± 3.571	6.16 ± 4.153	-2.079	0.040
No. of oocytes fertilized	2.81 ± 2.844	4.78 ± 3.643	-5.317	0.000
Fertilization rate, %	52.63(320/608)	77.64(545/702)	90.813	0.000
No. of cleavage embryos	2.55 ± 2.677	4.50 ± 3.605	-5.625	0.000
Cleavage rate, %	90.94(291/320)	94.13(513/545)	3.312	0.077
No. of top-quality embryos	0.48 ± 0.790	1.24 ± 1.626	-4.984	0.000
Top-quality embryos rate, %	18.90(55/291)	27.49(141/513)	7.423	0.006
No. of transferable embryos	1.08 ± 1.390	2.07 ± 2.315	-5.13	0.000
Transferable embryos rate, %	42.27(123/291)	46.00(236/513)	1.049	0.306
No. of embryos frozen	0.76 ± 1.046	1.54 ± 1.838	-5.114	0.000
Blastocyst formation rate, %	23.53(28/119)	23.87(53/222)	0.005	0.943
Cancellation rate per cycle, %	45.61(52/114)	28.95(33/114)	6.772	0.009

COS, controlled ovarian stimulation; MII, metaphase II; ICSI, intracytoplasmic sperm injection; 2PN, two pronuclei; AOA, artificial oocyte activation; positive number/total number in brackets.

### Embryological characteristics in previous standard ICSI cycles and AOA cycles

The stimulation protocols adopted in both groups were comparable. No significant differences were found in the number of oocytes obtained between the two groups. However, the numbers of oocytes fertilized, cleavage embryos, top-quality embryos, transferred embryos and frozen embryos were significantly higher in the AOA group than in the control group. Furthermore, the cycle cancellation rate, overall fertilization rate and top-quality embryo rate in the AOA group were significantly higher than those in the control group (P< 0.001). However, the cleavage rate, the transferable embryo rate, and blastocyst formation rate were comparable between the two groups (P>0.05) (**Table 2**).

### Pregnancy outcomes in previous standard ICSI cycles and AOA cycles

During the study period, women in the AOA group and women in the control group had undergone 84 and 60 embryo transfer cycles, respectively. The proportions of fresh transfer and FET were similar between the two groups (p>0.05). The clinical pregnancy rate per transfer (41.67% versus 8.33%, P < 0.001), clinical pregnancy rate per patient (30.7% versus 4.39%, P < 0.001) and implantation rate (34.59% versus 6.02%, P < 0.001) were significantly higher in the AOA group than in the control group, whereas the miscarriage rate in the AOA group was significantly lower than that in the control group (17.14% versus 100%, p< 0.001). Furthermore, the live birth rate per transfer (30.95%) and live birth rate per patient (28.95%) were greatly improved after AOA treatment compared to those in the control group (**Table 3**).

**Table 3:** Comparison of pregnancy outcomes between previous standard ICSI cycles and AOA cycles.

	Control group	AOA group	*t/χ ^2^/Z* value	*P* value
Total number of transfer cycles	60	84		
Fresh transfer (%)	25(15/60)	26.19(22/84)	0.026	0.872
FET (%)	75(45/60)	73.81(62/84)	0.026	0.872
No. of embryos transferred	0.73 ± 1.071	1.18 ± 1.151	-3.538	0.001
Clinical pregnancy rate per transfer (%)	8.33(5/60)	41.67(35/84)	19.385	0.000
Clinical pregnancy rate per patients (%)	4.39(5/114)	30.70(35/114)	27.287	0.000
Implantation rate (%)	6.02(5/83)	34.59(46/133)	32.985	0.000
Miscarriage rate (%)	100(5/5)	17.14(6/35)	15.065	0.000
Live birth rate per transfer (%)	0	34.52(29/84)		
Live birth rate per patients (%)	0	25.44(29/114)		

FET, frozen-thawed embryo transfer; positive number/total number in brackets.

### Subgroup analysis based on the cause of infertility

**Figure 2:**
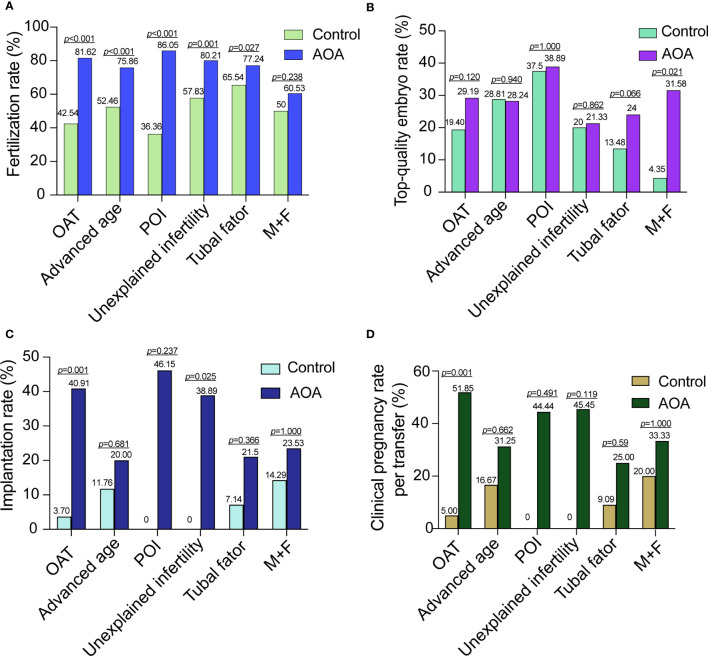
Comparison of activation effects in AOA subgroups based on the cause of infertility. **(A)** Comparison of fertilization rates between AOA subgroups and control groups. **(B)** Comparison of the top-quality embryo rate between AOA subgroups and control groups. **(C)** Comparison of the implantation rate between AOA subgroups and control groups. **(D)** Comparison of the clinical pregnancy rate between AOA subgroups and control groups. Control, previous ICSI cycles; *AOA*, artificial oocyte activation; *OAT*, oligoasthenoteratozoospermia; *POI* patients with primary ovarian insufficiency; *M+F* patients with male and female factors.

Subgroup analysis was performed into six categories according to the cause of infertility. The fertilization rates of each subgroup were significantly increased compared with those in their corresponding previous ICSI cycles without AOA except for patients in the M+F group. However, there was no significant difference in the top-quality embryo rate among all the subgroups except for the M+F group. The implantation rates were significantly increased in the OAT and unexplained infertility subgroups. The pregnancy rate was significantly improved in the OAT patients. For the OAT patients, the fertilization rate, implantation rate and clinical pregnancy rate were significantly increased, but no improvement was found in the top-quality embryo rate. In the unexplained infertility group, the fertilization rate and implantation rate were significantly increased after AOA, but no significant difference was found in the top-quality embryo rate and clinical pregnancy rate. For the M+F patients, the top-quality embryo rate was significantly increased after AOA, but no significant improvement was found in the top-quality embryo rate, implantation rate or clinical pregnancy rate. For the advanced age, POI and tubal factor patients, the fertilization rate was also significantly increased, but no significant increases were found in the top-quality embryo rate, implantation rate, and clinical pregnancy rate per patient after AOA treatment compared with their previous ICSI cycle (**Figure 2**).

### Subgroup analysis based on the reason for AOA

**Figure 3:**
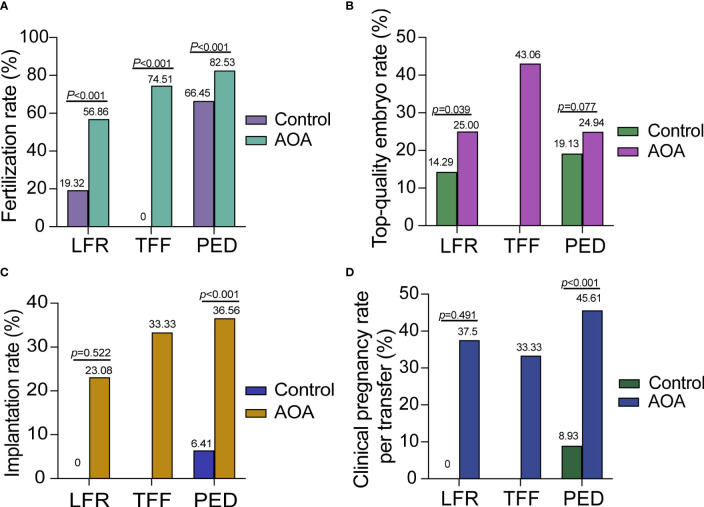
Comparison of activation effects in AOA subgroups based on the reason for AOA. **(A)** Comparison of fertilization rates between AOA subgroups and control groups. **(B)** Comparison of the top-quality embryo rate between AOA subgroups and control groups. **(C)** Comparison of the implantation rate between AOA subgroups and control groups. **(D)** Comparison of the clinical pregnancy rate between AOA subgroups and control groups. *Control* previous ICSI cycles; *AOA* artificial oocyte activation; *LFR* low fertilization; *TFF* total fertilization failure; *PED* poor embryo development.

Subgroup analysis was performed in three groups based on the reason for AOA. The fertilization rates of each subgroup were significantly increased compared with those in their previous ICSI cycle without AOA; however, there was no significant difference in the top-quality embryo rate. In the PED group, the fertilization rate, implantation rate and clinical pregnancy rate were highly increased after AOA, and the top-quality embryo rate was increased slightly after AOA treatment compared with their previous ICSI cycle without AOA but did not reach statistical significance. In the TFF and LFR groups, the fertilization rate was greatly improved, but no data on implantation rates and the clinical pregnancy rate were shown in the control group because no embryos could be transferred in these two groups (**Figure 3**).

### The neonatal outcomes in the AOA group

In the AOA group, 21 singletons and 7 twins among 35 babies were born. All newborns, including 15 boys and 20 girls, had no major birth defects or congenital malformations. Only one baby was born with an atrial septal defect in preterm with gestational weeks of 28 w+4 d. The other neonatal characteristics in the AOA group are shown in **Table 4** in terms of gestational weeks, body length, birth weight, low birth weight and preterm birth rate.

**Table 4:** Neonatal characteristics in the AOA group.

Item	Value
Birth babies	35
Baby’s gender	
Male, n	15
Female, n	20
Singletons, n	21
Twins, n	7
Gestational weeks (wk)	37.42 ± 3.200
Body length (cm)	48.26 ± 3.883
Birth weight (g)	2894.57 ± 773.143
Low birth weight rate (%) (<2,500 g)	28.57(10/35)
Preterm birth rate (%) (<37 wk)	25.71(9/35)
Birth defect rate (%)	2.86(1/35)

## Discussion

In this study, we demonstrated that AOA with ionomycin can improve the reproductive outcome of patients with previous total ICSI fertilization failure and poor embryo development.

Our results indicated that AOA treatment significantly improved the fertilization rate, implantation rate, clinical pregnancy and live birth rates per patient compared with previous ICSI cycles, which is in accordance with previous reports (19–21). The evidence suggested that AOA can improve the fertilization rate and the developmental ability of embryos and potentially provide more good-quality embryos. Additionally, some studies have also demonstrated that AOA leads to better cumulative pregnancy and live birth rates. However, one study (22) indicated that although AOA can improve the fertilization rate, it might not improve the developmental ability of embryos and pregnancy outcomes. This discrepancy might be derived from the different activation methods used. In that study, double ionophores were applied in cases with previous failed/low fertilization or poor embryo development, and patients with a previous single ionophore were used as the control group.

ICSI is the most effective method to treat male infertility, but complete fertilization failure will still occur occasionally in a few patients. Abnormal calcium oscillations after fertilization may be the principal mechanism for poor prognosis in patients with TFF. In this study, we found that the fertilization rate, implantation rate and clinical pregnancy rate were significantly increased in patients with TFF and LFR compared with previous ICSI cycles. AOA treatment with ionomycin can change the calcium oscillation pattern and improve the fertilization rate and clinical outcome of these patients. In the past 20 years, the application of AOA has successfully solved many infertility cases due to OAF caused by sperm factors, and Ca^2+^ ionophores have been successfully applied in cases of complete globozoospermia (23) or severe male factor infertility (24). Those results are consistent with our study, couples with OAT can benefit from ICSI-AOA in terms of higher fertilization rate, which in turn increases the number of available embryos and may improve the implantation and pregnancy rates. Recent researches found novel sperm-related oocyte activators, which showed that the ACTL7A/PLCZ1 mutation and DPY19 deletion decreased the expression of functional PLCζ, resulting in acrosomal detachment and oocyte activation failure associated with male infertility, and ICSI-AOA could rescue TFF in individuals with the ACTL7A/PLCZ1/DPY19 pathogenic variants (9, 25, 26). However, ICSI-AOA is not always beneficial for patients with previous LFR and a suspected oocyte-related activation deficiency (27), except Vanden Meerschaut F et al. reported that ICSI-AOA was very efficient in patients with a suspected oocyte-related activation deficiency and previous TFF after conventional ICSI. Our data demonstrated that the implantation rate, top-quality embryo rate and clinical pregnancy rate were not significantly different in the LFR group compared with previous ICSI cycles. AOA can cause calcium oscillation in oocytes, thereby improving fertilization rates in patients with LFR but may not improve embryo development and pregnancy outcomes in these patients.

Based on our study, we found that AOA treatment can improve the embryo development and reproductive outcome of patients with poor embryo development. This result is consistent with other studies, which demonstrated that AOA treatment can overcome the developmental incompetence of embryos and is an additional indication for ionophore treatment (14, 28). It was reported that the different frequencies and patterns of calcium oscillations could affect the induction of oocyte activation and embryo development (29). The calcium-binding proteins and intracellular calcium channels in the human endoplasmic reticulum that regulate inositol 1,4,5-trisphosphate receptors (IP3Rs) play an important role in the regulation of calcium signalling during oocyte maturation, fertilization, and early embryo development, and calcium signalling is a key factor in early embryo development (30). Therefore, AOA with calcium ionophores enhances calcium oscillations and may have beneficial effects on embryo development. However, one study on sibling oocytes showed that AOA using ionomycin 1 h after ICSI did not benefit the early or late development of embryos derived from patients with a history of embryo developmental problems (31). Admittedly, oocyte activation deficiencies are not the only reason for poor embryo development. Sperm DNA damage, oocyte abnormalities in structural proteins and mitochondria, or transcription factors may also contribute to poor prognosis of embryos (32), and none of them can be rescued by AOA.

We also observed that there was no significant difference in the top-quality embryo rate, embryo implantation rate or clinical pregnancy rate per patient in the advanced age and POI groups compared with the control group. These findings indicated that OAF might not be the main factor that affects infertility in advanced age and POI women. Problems with oocyte-derived and deficiencies in downstream signalling pathways may be the main cause of infertility in women of advanced age and POI (33). Furthermore, age-related defects may lead to mitochondrial DNA alignment, accompanied by mitochondrial dysfunction and changes in the gene expression profile (34). However, these studies suggested that AOA may not improve embryo development with advanced age and POI patients but may improve fertilization rates and thus potentially provide more transferable embryos (20). Vanden Meerschaut et al. also reported some cases of oocyte-related OAD, which could be overcome by AOA (27). In our study, the implantation rate was improved in patients with unexplained infertility, and the top-quality rate was also increased in patients with M+F.

We also found that the miscarriage rate in the AOA group was significantly lower than that in the control group. A prospective study demonstrated that the miscarriage rate was decreased in OAT, PCOS and unexplained infertility patients, which was in line with our study (35). Calcium oscillation is a landmark initial event in oocyte activation, and AOA can generate a single transient calcium oscillation, which may alter the activities of specific proteins and enzymes downstream of the Ca^2+^-regulated pathway and may also have a profound impact on gene expression and development at the later stage of embryos.

Although AOA can improve the treatment outcome of patients with fertilization failure, as a new technology in the assisted reproduction field, its safety still needs more in-depth study. At present, most reports believe that AOA technology will not increase the probability of embryo chromosome abnormalities or the risk of foetal birth defects. Miller et al. compared 83 offspring born with AOA and 595 offspring born after ICSI and demonstrated that there was no significant difference between the two groups in the rate and type of birth defects, birth weight, gestational age, and foetal sex of the offspring (36). A meta-analysis also confirmed that there was no significant difference in congenital defects or types of congenital defects between ICSI-AOA and traditional ICSI (37). These reports were consistent with our study, which showed no statistically significant differences in the birth defect rate, birth weight, gestational age, preterm birth rate, early-neonatal death rate, and foetal sex ratio between the activation and control groups. However, most current studies all have small sample sizes, and we only followed up on early foetal health. Moreover, another study revealed that the application of high concentrations of ionomycin after mouse sperm ICSI increased the frequency and amplitude of Ca^2+^ release, affecting mitochondrial energy metabolism, increasing reactive oxygen species (ROS) and decreasing ATP, and blastocyst formation (38). Furthermore, the impact of AOA on the gene expression and epigenetics of early human embryos is still lacking large amounts of research, and thus, further studies are needed to evaluate the long-term effect of AOA on offspring.

There are still some limitations in our study. First, we performed a retrospective self-controlled study. The previous ICSI cycles of patients served as the control group, and ICSI-AOA was performed in the second cycle based on the previous condition of patients. Patients may not use the same regimen twice. The health status of patients may be improved by medications in the second cycles, and the number and quality of oocytes retrieved may be improved by adjusting the ovulation stimulation protocol. Undoubtedly, the best control group would comprise patients undergoing ICSI-AOA versus patients undergoing ICSI only, and the reproductive outcomes can be compared between the two groups. The reason why we did not undergo a second cycle without AOA was that once the patients had already failed in the first ICSI cycle and exhibited fertilization failure or poor embryo development, they usually asked for something to change in the second cycle, and the physicians may probably recommend undergoing ICSI-AOA; thus, we lack a control group who are in a second cycle without AOA. Second, there may be some potential bias and confounders that cannot be excluded, and the POI, unexplained infertility and mixed factor infertility subgroups only had a small number of patients in each group; thus, the results should be interpreted with caution. Although the existing evidence was probably at a low level, this study might shed light on further research on AOA treatment, and another prospective, multicentre study with a larger sample size and parallel control would be needed in the future to provide a clearer answer.

In conclusion, our study demonstrated that AOA treated with ionomycin can obtain better reproductive outcomes in infertile couples with previous fertilization failure and poor embryo development. Subgroup analysis showed that couples with male/female factors and unexplained infertility may also benefit from AOA treatment in terms of improving fertilization rate and embryo development potential. However, before such procedures can be widely used in clinical practice, it is necessary to determine more physical agents and protocols that better mimic physiological Ca^2+^ release and conduct further research to evaluate the efficiency and safety of such treatments. At present, AOA should still be limited to application in patients with appropriate indications.

## Data availability statement

The raw data supporting the conclusions of this article will be made available by the authors, without undue reservation.

## Ethics statement

The studies involving humans were approved by Medical Ethics Committee of Shanghai First Maternal and Infant hospital (KS23210). The studies were conducted in accordance with the local legislation and institutional requirements. The participants provided their written informed consent to participate in this study.

## Author contributions

JR was involved in study design, execution, data analysis, project administration, roles/writing-original manuscript. JP and SL were involved in data collection and management ZC was involved in methodology, review and editing, critical discussion. XT was involved in review and editing, supervision critical discussion, and final approval of the manuscript. All authors read and approved the final manuscript. All authors contributed to the article and approved the submitted version.
